# Impact of sex and serum lipids interaction on working memory: A large‐scale brain networks study

**DOI:** 10.1002/brb3.3054

**Published:** 2023-05-10

**Authors:** Shujun Zhang, Xuezhen Li, Zhanguo Sun, Yueqin Chen, Yongqiang Yu

**Affiliations:** ^1^ Department of Radiology Affiliated Hospital of Jining Medical University Jining China; ^2^ Department of Radiology The First Affiliated Hospital of Anhui Medical University Hefei China

**Keywords:** large‐scale brain network, serum lipids, sex, working memory

## Abstract

**Backgrounds:**

Previous studies have demonstrated that both serum lipid levels and sex are crucial factors associated with individual cognition. However, the impact of sex and serum lipid interaction effects on the brain and cognition remains largely unknown. This study aimed to explore the underlying neural mechanisms among sex, serum lipids, and cognition using large‐scale brain networks.

**Methods:**

Resting‐state functional MRI data were collected from 157 young healthy adults. Independent component analysis was used to examine large‐scale inter‐ and intra‐network functional connectivity (FCs). Peripheral venous blood samples were collected to measure serum lipid levels. The three‐back task was employed to assess cognition (i.e., working memory). General linear model, correlation, and mediation analyses were conducted to examine the interaction effects of sex and serum lipids on large‐scale brain networks and their relationship with working memory.

**Results:**

We found that inter‐network connectivity with the executive control network at its core was more susceptible to sex and triglyceride interaction effects. The intra‐network connectivity in the dorsal attention networks (DANs), lateral visual networks, and anterior default mode networks was influenced by the interaction effects of sex and total cholesterol (TC)/low‐density lipoprotein cholesterol. Specifically, correlations between serum lipids and affected brain networks were found to be sex‐specific. In addition, higher intra‐network FC in the right inferior parietal (R‐IPL) of the DAN correlated with a longer three‐back reaction time in females. More importantly, the relationship between serum TC levels and three‐back reaction time was mediated by intra‐network connectivity in the R‐IPL of the DAN.

**Conclusions:**

Our findings describe the impact of sex and serum lipid interaction effects on large‐scale brain networks, as well as on cognitive function. Our data suggest that sex‐specific usage of serum lipids or brain networks would be beneficial for monitoring and therapy in dyslipidemia‐related cognition decline.

## Graphical abstract

Sex and serum lipids interaction affect large‐scale brain network connectivity. Internetwork connectivity with the executive control network at its core was more susceptible to sex and triglyceride interaction. The relationship between serum total cholesterol and 3‐back reaction time was mediated by the intranetwork connectivity of dorsal attention networks in female.

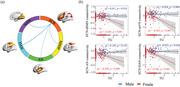

## 1 INTRODUCTION

Previous studies have demonstrated that serum lipid levels are associated with individual differences in cognition, such as working memory (Wang et al., 2020; Xia et al., 2015), executive control, and sustained attention (Gendle et al., 2008). Numerous cross‐sectional and longitudinal studies have consistently shown that higher serum total cholesterol (TC) (Gendle et al., 2008; Solomon et al., 2009; Xia et al., 2015), triglycerides (TG) (Parthasarathy et al., 2017), and low‐density lipoprotein cholesterol (LDL‐C) (Bates et al., 2017; Power et al., 2018) levels are associated with poor cognitive performance, whereas higher serum high‐density lipoprotein cholesterol (HDL‐C) levels predict the better maintenance of cognitive function (Bates et al., 2017; Reynolds et al., 2010; Sun et al., 2019). In humans, the brain has the second‐highest lipid content after adipose tissue, accounting for 50% of its dry weight (Hornemann, 2021). High lipid content plays a crucial role in the brain, because lipids provide structural integrity and modulate the fluidity of brain neuronal cells. Lipid dysregulation has been associated with the etiology and progression of neurodegeneration and other neurological pathologies (Pfrieger, 2003; Yoon et al., 2022). Therefore, lipids are emerging as important potential targets for the early diagnosis and prognosis of neurological diseases. Recent advances in neuroimaging technologies, especially brain functional connectivity (FC), have facilitated studies examining the relationship between serum lipids and the brain in both normal and disease‐affected populations. For example, cholesterol was found to accelerate the impact of age on neural trajectories by disrupting FC in circuits implicated in integrative processes and behavioral control (Spielberg et al., 2017). High serum cholesterol has been associated with the disruption of FC in the salience network in non‐demented elderly persons (Zhang et al., 2016). Disruption of the resting‐state connectivity in the hippocampus and middle frontal gyrus may mediate the relationship between poorly controlled cholesterol, impaired attention, and executive function in type 2 diabetes mellitus (T2DM) patients (Xia et al., 2015).

Similar to serum lipids, biological sex is also an important factor that can impact the cognitive function of humans. Sex‐specific differences have been identified not only in advanced neurocognitive processes, such as attention (Bangasser et al., 2019), verbal working, and spatial memory (Chen et al., 2020; Voyer et al., 2021), but also in cognitive development and psychiatric disease (Kaczkurkin et al., 2019). Earlier studies suggested that sex‐specific cognitive differences were due to differences in the structure and function of the brain in males and females (de Lacy et al., 2019; Gur & Gur, 2017; Ingalhalikar et al., 2014; Sang et al., 2021; van Eijk et al., 2021). Indeed, the brain network not only exhibits sexual dimorphism but also plays a vital role in mediating sex‐related cognitive changes (Li et al., 2021; Li et al., 2022; Murray et al., 2021; Tunc et al., 2016; Xu et al., 2020; Zhao et al., 2021; Zhao et al., 2020). In addition, sex‐specific differences in serum lipid profiles have been frequently reported in healthy aged individuals and have been associated with worse cognitive performance (Lu et al., 2017). Previous studies have suggested that physiological doses of sex steroid hormones can affect serum lipid and lipoprotein levels in humans (Engelberg & Glass, 1955). However, the interactive role of sex‐specific brain network mechanisms and specific lipids on cognitive performance remains unclear.

The human brain is a complex system consisting of multiple distinct and interacting functional networks that subserve different functions (De Luca et al., 2006; Power et al., 2011). Each functional network is composed of several brain regions with a high degree of consistency in signal change over the course of resting‐state functional MRI (rs‐fMRI). Different networks display diverse activity patterns. These functional networks can be automatically identified by independent component analysis (ICA) of rs‐fMRI data, a useful method enabling data‐driven, exploratory investigation of temporal correlations among brain regions at rest (Calhoun et al., 2001; van de Ven et al., 2004). This approach has been broadly applied to explore the underlying relationships between cognition and large‐scale functional organization (inter‐ and intra‐network FCs) in normal and abnormal brains (Wang et al., 2020; Zhang et al., 2021). Inter‐network FC reflects the information exchange capability between different regions, whereas intra‐network FC reflects information specialization within specific functional networks (Liao et al., 2017). Abnormal integration and separation of information between brain networks have been examined in some psychiatric disorders, which can potentially lead to cognitive changes (Wei et al., 2022; Yang et al., 2016; Zhu et al., 2016). However, as previous studies mainly focused on the relationship between sex or lipids on cognition in specific brain regions or networks, little is known about the roles of inter‐ and intra‐network FCs in the interaction effects of sex and serum lipids on cognition.

Based on these earlier studies, we sought to determine how large‐scale brain networks mediate the interaction effects of sex and serum lipids on cognitive function by performing an rs‐fMRI analysis with ICA and quantifying large‐scale inter‐ and intra‐network FCs in 157 healthy adults. Peripheral venous blood samples were collected to measure serum lipid levels. The three‐back task was employed to assess working memory. Using these data, we first sought to determine the inter‐ and intra‐network FCs that were influenced by the interaction effects of sex and serum lipids. Second, we aimed to assess the associations of affected brain networks with working memory in males and females separately. Finally, we attempted to determine the mediative role of these identified FC markers in accounting for the associations between serum lipids and working memory in males and females separately. Based on our findings, we hypothesize that serum lipids affect cognition through large‐scale brain networks in a sex‐specific manner.

## 2 MATERIALS AND METHODS

### 2.1 Participants

The study included 157 healthy young adults who were recruited by advertisement. All participants fulfilled the following inclusion criteria: Han Chinese; right‐handed; and age from 18 to 30 years. Exclusion criteria included neuropsychiatric or severe somatic disorders, a history of alcohol or drug abuse, regular smoker, recent medication (e.g., antibiotics or sedative hypnotics) within the past month, systemic inflammation, severe gastrointestinal disorders, pregnancy, menstruating females, MRI contraindications, and a family history of psychiatric illness among first‐degree relatives. The MINI‐International Neuropsychiatric Interview (M.I.N.I.) and Alcohol Use Disorders Identification Test (AUDIT) were used to exclude participants. The participants’ dietary habit information was collected using the Dietary Nutrition and Health Questionnaire (DNHQ), which is a self‐report questionnaire that includes 50 items. Total scores range from 50 to 200 points, with a lower score reflecting better dietary habits. This study was approved by the ethics committee of The First Affiliated Hospital of Anhui Medical University. Written informed consent was obtained from all participants after providing them with a complete description of the study. Detailed data of the participants are listed in Table 1.

**TABLE 1:** Demographic and cognitive characteristics of the study participants

Characteristics	Males	Females	*t* Value	*p* Value
Number of subjects	80	77	–	–
Age (years)	21.80 ± 2.39	22.87 ± 2.34	−2.84	.005
Education (years)	15.26 ± 1.93	16.32 ± 1.77	−3.60	<.001
FD (mm)	0.13 ± 0.06	0.11 ± 0.03	2.35	.02
DNHQ	102.73 ± 10.08	101.12 ± 10.78	0.97	.34
C‐reactive protein	0.94 ± 1.07	1.21 ± 3.28	−0.68	.50
Anti‐O antibody	108.75 ± 125.04	90.92 ± 98.83	0.99	.32
Rheumatoid factor	3.64 ± 3.48	3.47 ± 3.64	0.28	.78
**Serum lipid parameters**				
TC (mmol/L)	4.03 ± 0.69	4.13 ± 0.82	−0.89	.37
TG (mmol/L)	1.12 ± 0.62	0.86 ± 0.38	3.29	.001
HDL‐C (mmol/L)	1.33 ± 0.26	1.69 ± 0.31	−7.76	<.001
LDL‐C (mmol/L)	2.28 ± 0.63	2.13 ± 0.71	1.383	.17
**Three‐back task performance**				
Three‐back reaction time	761.66 ± 179.97	776.37 ± 171.04	−0.53	.60
Three‐back accuracy	0.73 ± 0.16	0.72 ± 0.16	0.39	.70

*Note*: Data are presented as mean ± SD.

Abbreviations: DNHQ, Dietary Nutrition and Health Questionnaire; FD, frame‐wise displacement; HDL‐C, high‐density lipoprotein cholesterol; LDL‐C, low‐density lipoprotein cholesterol; SD, standard deviation; TC, total cholesterol; TG, triglyceride.

### 2.2 Blood sampling and serum lipid measurement

Blood samples were collected within 1 day before or after MRI examination. After an overnight fasting period, peripheral venous blood samples (2 mL) were collected from all participants the following morning. Samples were centrifuged to separate the serum at 3000 rpm for 10 min at room temperature, and lipid profile analysis was carried out immediately on the fresh serum. Serum TC, TG, and HDL‐C levels were measured in an automated clinical analyzer (Roche cobas 8000). Serum LDL‐C levels were estimated using the Friedewald equation.

### 2.3 Working memory assessment

The letter three‐back task was performed on a computer to assess working memory using E‐Prime 2.0 software (http://www.pstnet.com/eprime.cfm) (Owen et al., 2005). During the task, each participant viewed a series of sequentially presented letters, with each letter stimulus presented for 200 ms and an interval of 1800 ms between stimuli. Participants were instructed to press a button on the right with their middle finger if the letter appearing on the screen was identical to the one displayed three letters earlier. Otherwise, they pressed a button on the left with their index finger. The task consisted of 60 trials. Prior to the formal test, participants received verbal instructions and underwent a practice test. Working memory performance was measured using two metrics: accuracy and the mean reaction time of correct responses. An increase in accuracy and reduction in mean reaction time signified a better working memory.

### 2.4 MRI data acquisition

MRI scans were obtained using a 3.0 Tesla MR system (Discovery MR750w, General Electric, Milwaukee, WI, USA) with a 24‐channel head coil. Earplugs were used to reduce scanner noise, and tight but comfortable foam padding was used to minimize head motion. High‐resolution 3D T1‐weighted structural images were acquired by employing a brain volume (BRAVO) sequence with the following parameters: repetition time (TR) = 8.5 ms; echo time (TE) = 3.2 ms; inversion time (TI) = 450 ms; flip angle = 12°; field of view (FOV) = 256 mm × 256 mm; matrix size = 256 × 256; slice thickness = 1 mm, no gap; 188 sagittal slices. Resting‐state blood‐oxygen‐level‐dependent (BOLD) fMRI data were acquired using a gradient‐echo single‐shot echo planar imaging (GRE‐SS‐EPI) sequence with the following parameters: TR = 2000 ms; TE = 30 ms; flip angle = 90°; FOV = 220 mm × 220 mm; matrix size = 64 × 64; slice thickness = 3 mm, slice gap = 1 mm; 35 interleaved axial slices; 185 volumes. All images were visually inspected to ensure that only images without visible artifacts (e.g., ghosting artifacts arising from subject movement and pulsating large arteries, metal artifacts, susceptibility artifacts, and blooming artifacts) were included in subsequent analyses. None of the participants were excluded for visually inspected imaging artifacts.

### 2.5 fMRI data preprocessing

Resting‐state BOLD data were preprocessed using Statistical Parametric Mapping software (SPM12, http://www.fil.ion.ucl.ac.uk/spm) and data processing & analysis for brain imaging (DPABI, http://rfmri.org/dpabi) (Yan et al., 2016). The first 10 volumes for each participant were discarded to allow the signal to reach equilibrium and the participants to adapt to the scanning noise. The remaining volumes were corrected for the acquisition time delay between slices. Then, realignment was performed to correct the motion between time points. Head motion parameters were computed by estimating the translation in each direction and the angular rotation on each axis for each volume. All participants’ BOLD data were within the defined motion thresholds (i.e., translational or rotational motion parameters less than 2 mm or 2°). We also calculated frame‐wise displacement (FD), which indexes the volume‐to‐volume changes in head position. In the normalization step, individual structural images were first co‐registered with the mean functional image; then the transformed structural images were segmented and normalized to the Montreal Neurological Institute (MNI) space using a high‐level nonlinear warping algorithm, that is, the diffeomorphic anatomical registration through the exponentiated Lie algebra (DARTEL) technique (Ashburner, 2007). Finally, each filtered functional volume was spatially normalized to MNI space using the deformation parameters estimated during the above step and resampled into a 3‐mm cubic voxel. After spatial normalization, all data sets were smoothed with a Gaussian kernel of 6 × 6 × 6 mm^3^ full‐width at half maximum.

### 2.6 Independent component analysis

ICA was employed to parcellate the preprocessed fMRI data with the GIFT toolbox (http://mialab.mrn.org/software/gift/), and the number of independent components (*N* = 26) was estimated automatically by the software using the minimum description length criteria. Spatial ICA separates the participant data into linear mixtures of spatially independent components that exhibit a unique time course profile. Detailed quality control and preprocessing procedures are described in our previous study (Zhang et al., 2021). Finally, 14 functional networks out of 26 independent components were obtained (Figure S1): anterior and posterior default mode networks (aDMN and pDMN); executive control network (ECN); left and right frontoparietal networks (lFPN and rFPN); salience network (SN); dorsal and ventral attention networks (DAN and VAN); dorsal and ventral sensorimotor networks (dSMN and vSMN); auditory network (AN); and medial, lateral, and posterior visual networks (mVN, lVN, and pVN).

### 2.7 Statistical analysis

The statistical descriptive analyses of demographic, serum lipid levels, and behavioral data were conducted using the SPSS 23.0 software package (SPSS, Chicago, IL, USA). We adopted a multistage approach to analyze the data of sex and serum lipids (TC, TG, LDL‐C, and HDL‐C) interaction effects, large‐scale brain networks (inter‐ and intra‐network FCs), and working memory (three‐back accuracy and reaction time). Sex was coded as male = 1 and female = 2. First, we tested for the sex and serum lipids interaction effect on inter‐ and intra‐network FCs. For inter‐network functional analysis, a general linear model was utilized to examine this effect with age and FD as nuisance covariates. Multiple comparisons were corrected using the false discovery rate (FDR) method, with a corrected significance threshold of *p* < .05. For intra‐network functional analysis, all participants’ spatial maps for each functional network were initially entered into a random‐effect one‐sample *t*‐test. Brain regions were considered to be within each network if they met a height threshold of *p* < .05 corrected for multiple comparisons using a family‐wise error method. Next, the sex and serum lipids interaction effect on intra‐network FC was assessed in a voxel‐wise manner within each network using a general linear model with age and FD as nuisance variables. Multiple comparisons were corrected using the cluster‐level FDR method, resulting in a cluster‐defining threshold of *p* = .001 and a corrected cluster significance of *p* < .05. The partial correlation coefficients between serum lipid and inter‐ or intra‐network FCs were transformed into Fisher's *Z* scores and then compared between males and females (Nostro et al., 2017). Second, in cases of significant interaction effects, we further examined the association between affected brain networks and working memory using partial correlations adjusted for age, FD, and educational level in males and females separately. Finally, to determine whether the relationship between serum lipids and working memory was mediated by identified FC in a sex‐specific manner, mediation analysis (Figure S2) was performed using PROCESS macro (Honey et al., 2009). A detailed description of the mediation models can be found in the Supporting Information section.

## 3 RESULTS

### 3.1 The sex and serum lipids interaction effect on inter‐network FC and its relationship with working memory

**FIGURE 1:**
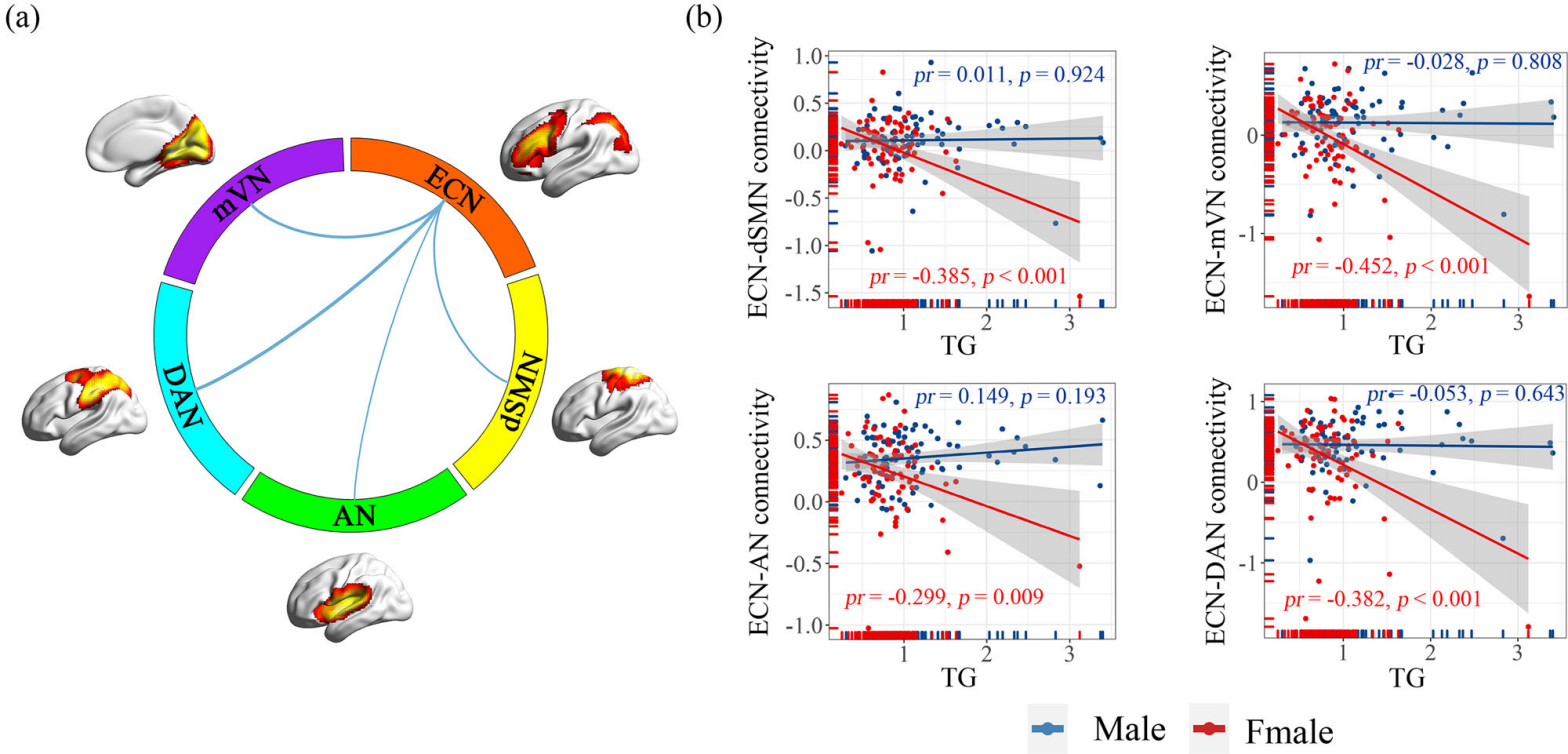
(A) Interaction effects of sex and serum lipids on inter‐network functional connectivity. Line thickness denotes magnitude of the slope in sex and serum lipids interaction on inter‐network functional connectivity. Cool colors represent negative correlations. (B) Scatter plots showing the significant correlation between affected inter‐network functional connectivity and triglyceride levels in females, but not males. AN, auditory network; dSMN, dorsal sensorimotor network; DAN, dorsal attention network; ECN, executive control network; mVN, medial visual network; TG, triglyceride.

A significant sex and TG interaction effect on inter‐network FC was observed (*p* < .05, FDR corrected; Figure 1). Specifically, we found negative interaction effects of sex and TG on the inter‐network connectivity between the ECN and dSMN (*F* = −3.439, *p* = .023), ECN and mVN (*F* = −4.150, *p* = .005), ECN and AN (*F* = −3.328, *p* = .025), and ECN and DAN (*F* = −3.602, *p* = .019). However, no significant interaction effects of sex and serum levels of other lipid parameters (i.e., TC, HDL‐C, and LDL‐C) were found on the inter‐network FC.

Next, we examined the effect of TGs on the inter‐network FC significantly affected by the sex and TG interaction effect in males and females separately (Figure 1 and Table S1). Partial correlation analysis showed that TG was negatively correlated with inter‐network connectivity between ECN and dSMN (partial correlation coefficient (*pr*) = −0.385, *p* < .001), ECN and mVN (*pr* = −0.452, *p* < .001), ECN and AN (*pr* = −0.299, *p* = .009), and ECN and DAN (*pr* = −0.382, *p* < .001) in females. However, no significant correlation between the inter‐network connectivity and TG was observed in males (all *p* > .05). Furthermore, the relationship between TG‐related inter‐network connectivity and working memory in females was not significant.

### 3.2 The sex and serum lipids interaction effect on intra‐network FC and its relationship with working memory

**FIGURE 2:**
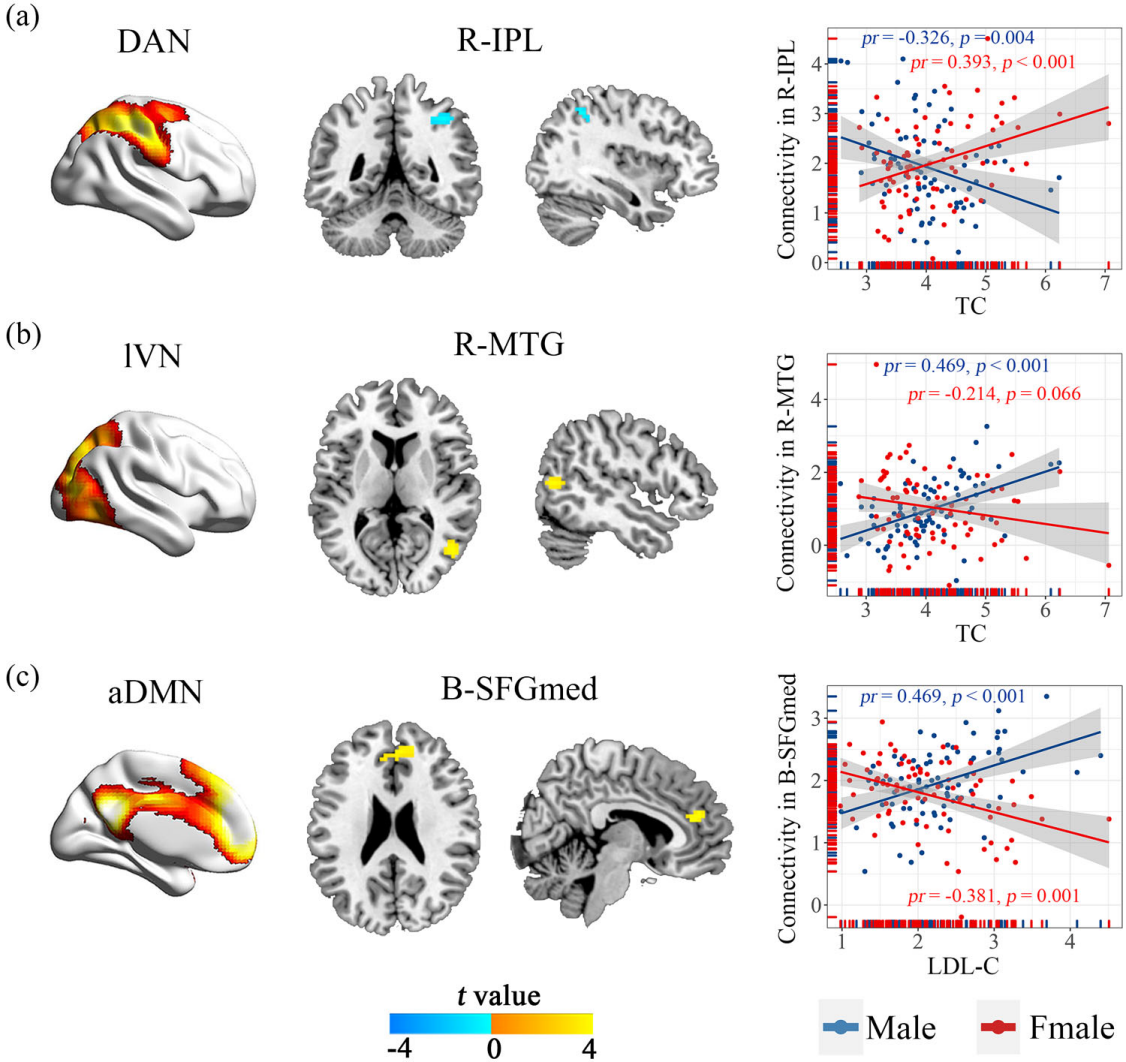
Interaction effects of sex and serum lipids on intra‐network functional connectivity (FCs). Scatter plots showing that the correlation between serum lipids and affected intra‐network FCs are sex‐specific. (A, B) The intra‐network FCs in the right inferior parietal lobe of the dorsal attention network and right middle temporal gyrus of the lateral visual network were influenced by the interaction effect of sex and total cholesterol.(C) The intra‐network FC in the bilateral medial superior frontal gyrus of anterior default mode networks was influenced by the interaction effect of sex and low‐density lipoprotein cholesterol. aDMN, anterior default mode networks; B, bilateral; DAN, dorsal attention network; IPL, inferior parietal lobe; LDL‐C, low‐density lipoprotein cholesterol; lVN, lateral visual network; MTG, middle temporal gyrus; R, right; SFGmed, medial superior frontal gyrus; TC, total cholesterol.

Voxel‐wise intra‐network FC analyses demonstrated that the connectivity within multiple functional networks was influenced by the interaction effect of sex and serum lipids (*p* < .05, cluster‐level FDR corrected; Figure 2). Specifically, the intra‐network FC in the right inferior parietal lobe (R‐IPL, cluster size = 31 voxels; peak MNI coordinates: *x*/*y*/*z* = 36/−51/48; peak *t* = −4.24) of the DAN and right middle temporal gyrus (R‐MTG, cluster size = 25 voxels; peak MNI coordinates: *x*/*y*/*z* = 48/−69/9; peak *t* = 4.02) of the lVN were influenced by the interaction effect of sex and TC (Figure 2A,B). The intra‐network FC in the bilateral medial superior frontal gyrus (B‐SFGmed, cluster size = 53 voxels; peak MNI coordinates: *x*/*y*/*z* = 3/42/21; peak *t* = 4.41) of the aDMN was influenced by the interaction effect of sex and LDL‐C (Figure 2C).

Next, we examined the relationship between serum lipids (TC and LDL‐C), and the intra‐network FCs significantly affected by the sex and serum lipids interaction effect in males and females separately. As shown in Figure 2 and Table S2, TC was negatively correlated with intra‐network connectivity in the R‐IPL in males (*pr* = −0.326, *p* = .004), whereas a positive correlation was found in females (*pr* = 0.393, *p* < .001). TC was positively correlated with intra‐network connectivity in the R‐MTG (*pr* = 0.469, *p* < .001) in males, whereas no correlation was observed in females (*pr* = −0.214, *p* = .066). LDL‐C was positively correlated with the intra‐network connectivity in the B‐SFGmed (*pr* = 0.469, *p* < .001) in males, whereas a negative correlation was found in females (*pr* = −0.381, *p* = .001).

**FIGURE 3:**
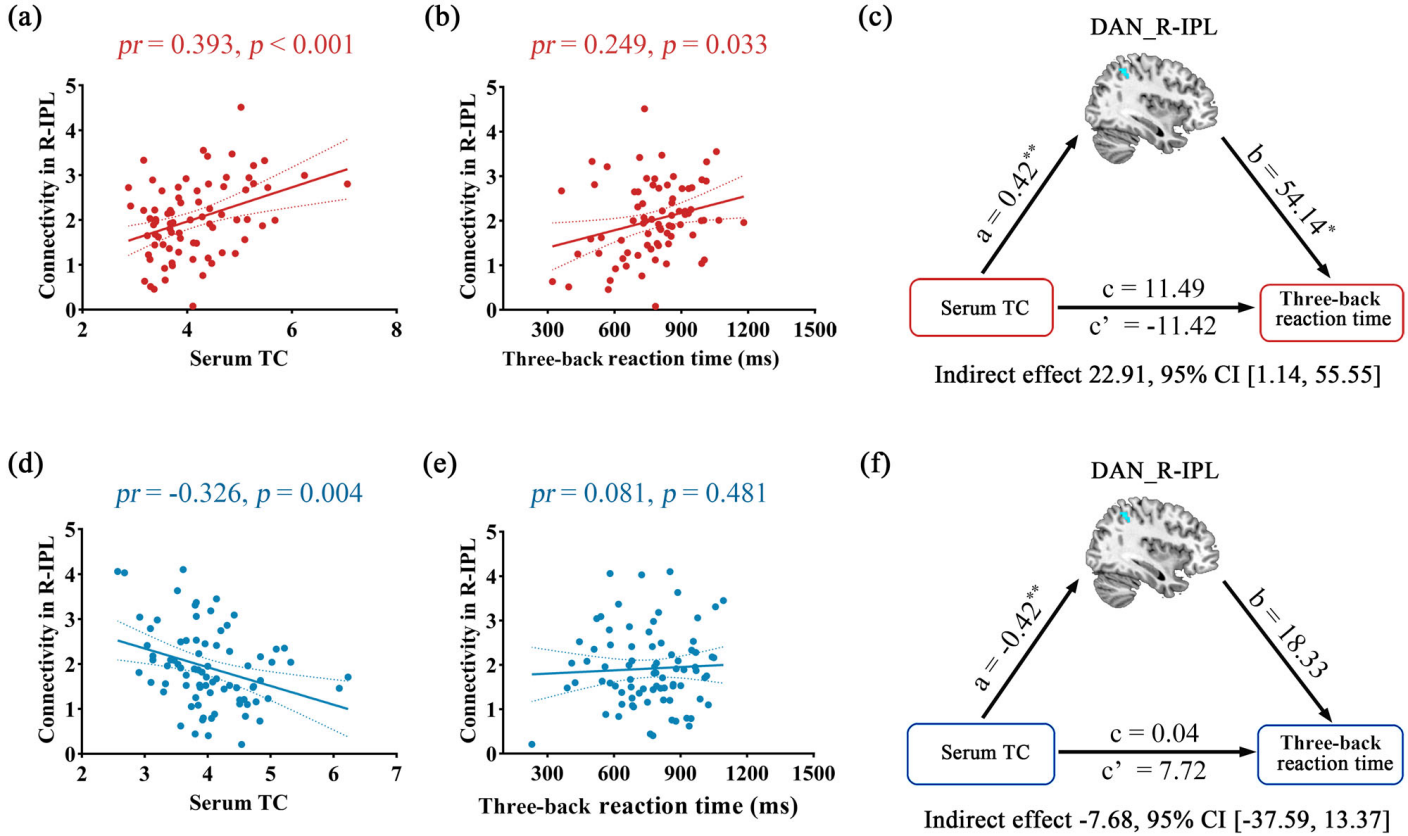
Scatter plots showing the correlation between intra‐network functional connectivity, serum total cholesterol (TC) levels, and three‐back reaction time in females (A, B) and males (D, E). (C, F) Mediation analyses between serum TC (X) and three‐back reaction time (Y) with intra‐network functional connectivity in the right inferior parietal (R‐IPL) of the DAN as the mediator (M). Path coefficients with *p* values (∗*p* < .05 and ∗∗*p* < .01, respectively). DAN, dorsal attention network; IPL, inferior parietal lobe; R, right.

The relationships between the intra‐network connectivity in the R‐IPL, R‐MTG, and B‐SFGmed and cognitive performance were further analyzed in males and females separately. Our data indicated that the three‐back reaction time was positively correlated with intra‐network connectivity in the R‐IPL of the DAN in females (Figure 3A,B), but not in males (Figure 3D,E). Further mediation analyses revealed that intra‐network connectivity in the R‐IPL significantly mediated the relationship between serum TC levels and three‐back reaction time in females (indirect effect = 22.91, SE = 14.16, 95% CI: 1.14, 55.55) (Figure 3C), but not in males (Figure 3F).

### 3.3 Sensitivity analysis

To test the possible effect of education level on our results, we included education level as an additional nuisance covariate in the analyses of the sex and serum lipids interaction effect on inter‐ and intra‐network FCs. As shown in Tables S3 and S4, our main results were preserved after additional adjustments for education level (*p* < .05). Furthermore, there are significant differences in serum TG levels between male and female subjects, which may influence the reliability of the sex and TG interaction effect. To ensure the robustness of our findings, we selected 70 male and 70 female participants from the total sample, ensuring that their TG levels were matched (*t* = 0.487, *p* = .627). We subsequently reevaluated the interaction effect of TG and sex on inter‐network FC, and our results remained consistent (Table S5). Moreover, our analysis has confirmed that the current sample size is sufficient to yield statistically significant results, as depicted in Figure S3.

## 4 DISCUSSION

Our study is the first to investigate the interaction effects of sex and serum lipids on cognitive function using large‐scale brain networks. Our analysis demonstrated that sex had an effect on the relationship between serum lipid levels and inter‐network FC in the ECN‐dSMN, ECN‐mVN, ECN‐AN, and ECN‐DAN and intra‐network FC in the R‐IPL, R‐MTG, and B‐SFGmed. We found that intra‐network connectivity in the R‐IPL significantly mediated the relationship between serum TC levels and the 3‐back reaction time in females, but not in males. Thus, our findings suggest that sex may be an important factor that needs to be considered when examining the relationship between serum lipid levels, brain, and cognition.

Extensive studies have revealed that serum lipids, as well as sex dimorphism, are involved in the onset and progression of different neuropsychiatric disorders, such as depression (Xu et al., 2021), first‐episode schizophrenia (Gjerde et al., 2021), cerebral small vessel disease (Yin et al., 2018), and Parkinson's disease (Seyfried et al., 2018). However, the neurobiological bases of the sex‐specific different contributions of lipids fractions on neuropsychiatric disorders are still unclear, probably due to the few studies available to date which have carried out sex‐stratified analysis. Although few studies have examined the brain network mechanisms through which blood lipids and sex affect behavioral performance including cognition, some efforts to determine the relationship between serum lipids and brain FC have been made. For example, stronger connectivity in the DMN and lower connectivity in the SN have been observed in nondemented elderly with high serum TC levels (Zhang et al., 2016). Poorly controlled cholesterol has been shown to impair FC in the hippocampus and middle frontal gyrus in T2DM patients (Xia et al., 2015). In addition, TGs have been shown to affect the inter‐network FC between SN and vSMN, which in turn impacts cognition (Wang et al., 2020). However, these earlier studies did not examine sex‐specific effects, which may impact the correlation between brain network connectivity and serum lipids. In the current study, we assessed the interaction effects of sex and serum lipid levels on brain networks using the ICA method, which facilitates a more thorough characterization of the whole‐brain functional connectome. We found that the functional exchange and integration between executive control and sensorimotor systems (i.e., the inter‐network FC in the ECN‐dSMN, ECN‐mVN, ECN‐AN, and ECN‐DAN) were preferentially associated with serum TG levels, and that these relationships displayed sex dimorphism—that is, negative correlation in females, and no correlation in males. The ECN is the core network among all the affected inter‐network functional connections and is primarily anchored in the prefrontal and lateral parietal regions and is thought to be associated with working memory and attention processes (Cai et al., 2021; Song et al., 2013). Filippi et al. (2013) examined the effects of sex on resting‐state functional networks and found that women displayed stronger connectivity in the frontotemporal regions and within attention and memory‐related networks. In addition, Banks suggested that elevated TG levels may compromise the blood–brain barrier transport of insulin and other hormones (Banks, 2012), thereby exerting a pro‐inflammatory effect, which may negatively impact cognitive performance. Knopp et al. (2006) reported that high TG levels exerted a worse cardiovascular outcome in women than men. Thus, it is possible that in cognitively impaired patients, hypertriglyceridemia exerts a more detrimental effect in women than in men. These findings are consistent with our study, which demonstrated that inter‐network connectivity with the ECN at its core is more susceptible to serum TG levels in females, which may further influence cognitive performance. In short, such findings may help explain our results, which found that the effect of TG on ECN‐related inter‐network FC was sex‐dependent. Our results, together with those of previous studies (Gjerde et al., 2021; Seyfried et al., 2018; Xu et al., 2021; Yin et al., 2018), suggest that alterations in inter‐network FC may be a potential network mechanism of lipid‐involved neuropsychiatric disorders that exhibit sex dimorphism.

Dense intra‐network connections increase local clustering and thus facilitate information specialization within a specific functional network. Thus, studies examining intra‐network temporal coherence may further our understanding of functional specialization (Liao et al., 2017). Previous voxel‐wise analyses of spatial maps revealed no significant correlations between serum lipid levels and intra‐network connectivity within any of the functional networks, when sex was considered to be a nuisance variable (Wang et al., 2020). However, the effect of sex on the relationship between serum lipid levels and variations in neuroimaging phenotypes should not be ignored. In the current study, we found that sex and serum lipid interaction effects affected the intra‐network FC in the DAN, lVN, and aDMN, further suggesting that sex is a key factor that needs to be considered when examining the relationship between serum lipids and brain networks. Previous studies have also shown that the relationship between serum lipid levels and cognition exhibited sex specificity. For example, in a cross‐sectional study of aging women, serum high‐density lipoprotein was found to be associated with better verbal learning and memory performance (Bates et al., 2017). Another 16‐year longitudinal study suggested that higher HDL‐C and lower TG levels predicted better maintenance of cognitive functions (including verbal ability and perceptual speed) in women than men (Reynolds et al., 2010). Interestingly, in the present study, we found sex‐specific differences in the association between lipid fractions and the intra‐network FC. In particular, a positive association between intra‐network FC in the R‐IPL of DAN and working memory was found only in women. Similarly, intra‐network FC in the DAN mediated the relationship between serum TG levels and working memory, but this was only observed in women. In contrast, an inverse association between lipid fractions and intra‐network FC was found in men. Recent studies have reported that activation or impairment of the DAN is associated with short‐term memory (Majerus et al., 2012) and memory coding (Mallas et al., 2021). Our study extends these findings by showing that the density of FC in the DAN is more susceptible to serum TC in females, and that changes in the degree of information specialization within DAN are related to working memory. Furthermore, the correlation between the intra‐network FC in the R‐MTG of the lVN with TC and the correlation between the intra‐network FC in the B‐SFGmed of the aDMN with LDL‐C were also found to have sex‐specific differences. The lVN is mainly implicated in the processing of visual information, whereas the DMN is thought to support internally oriented cognitive processes, including recalling autobiographical and episodic memories, envisioning the future, and making social inferences (Raichle et al., 2001). Our results may provide new perspectives on the treatment of lVN‐ and DMN‐related functional disorders, through sex‐specific serum lipid interventions. Furthermore, different strategies should be used to adjust serum lipid levels or lipid‐related brain networks in the treatment of psychiatric disorders associated with sex differences such as autism, which is more prevalent in men (Lai et al., 2015), and depression, which is more prevalent in women (Ferrari et al., 2013).

Here, we found that both the inter‐ and intra‐network FCs were influenced by the serum lipids and sex interaction effect and further affected cognition in a sex‐specific manner. This may be because estrogen and progesterone fluctuations throughout the menstrual cycle are not only related to brain networks such as transient reorganization and topological properties of functional brain networks (Liparoti et al., 2021; Mueller et al., 2021), but also to serum lipids. For example, estrogen has been associated with reduced LDL‐C levels and increased HDL‐C and TG levels (Engelberg & Glass, 1955; Gambrell & Teran, 1991; Rezvani et al., 2014). Another possible explanation is that higher levels of testosterone, and its stable concentration across the life span in males may be responsible for sex‐specific differences in the associations between serum lipids and large‐scale brain networks (Liu et al., 2015).

There are several limitations in the present study. First, our cross‐sectional design does not allow for inference to be drawn regarding causality. Nevertheless, due to our relatively large sample size, our findings can provide a useful springboard for further research. Second, as our study sample was selected from a group of educated young adults, our findings may not be generalizable to older populations with mental illness. Third, diet has a diverse effect on serum lipids (Li & Shi, 2017). However, as all participants enrolled in the current study fasted overnight prior to the collection of blood samples, diet should not significantly impact our results. Fourth, the lack of data on serum sex hormones is also an important constraint as, for example, estrogen and progesterone fluctuations over the menstrual cycle are associated with the brain network and serum lipid levels. However, it may be difficult to fully understand the effects of sex hormones in a clinical setting due to the complex regulatory mechanisms that are involved. Finally, in the current study, cognitive function was measured using a three‐back task test, which only assesses working memory. Future studies need to utilize a more comprehensive measure involving multiple cognitive domains.

To our knowledge, this is the first study that has specifically examined the interaction effects of sex and serum lipids on the relationship between large‐scale brain networks and cognition in healthy adults. Our findings suggested that interactions between sex and serum lipids may affect cognition through large‐scale inter‐ and intra‐network FCs. Our data may be of clinical relevance because they indicate that sex should be taken into account when using serum lipid levels or related‐brain networks to monitor treatment response during cognition decline. In addition, our results may facilitate the development of novel therapeutic strategies that are more tailored to the sex of the patient, thus increasing the likelihood of successful treatment.

## AUTHOR CONTRIBUTIONS

Shujun Zhang and Yongqiang Yu conceptualized and designed the study. Shujun Zhang was responsible for conducting the analyses, preparing the first draft of the manuscript, and preparing the manuscript for submission. Yongqiang Yu was responsible for obtaining funding for the study, supervising the analyses, and editing drafts of the manuscript. All authors contributed to and approved the final manuscript.

### PEER REVIEW

The peer review history for this article is available at https://publons.com/publon/10.1002/brb3.3054.

## Supporting information


**Figure S1**. Spatial maps of 14 selected functional networks.
**Figure S2**. Mediation analysis model.
**Figure S3**. Using G*Power software, a post hoc analysis was performed to determine the relationship between statistical power and sample size, our samples could detect with adequate power (>95%) based on an alpha=0.05, indicating that our sample size was adequate.
**Table S1**. Sex differences in the associations between triglyceride and inter‐network functional connectivity.
**Table S2**. Sex differences in the associations between serum lipid and intra‐network functional connectivity.
**Table S3**. Sex differences in the associations between triglyceride and inter‐network functional connectivity after additional adjustment for education level.
**Table S4**. Sex differences in the associations between serum lipid and intra‐network functional connectivity after additional adjustment for education level.
**Table S5**. Sex differences in the associations between triglyceride (TG) and inter‐network functional connectivity after matching the serum TG levels between male and female subjects.

## Data Availability

The data that support the findings of this study are available from the corresponding author upon reasonable request. The data are not publicly available due to privacy or ethical restrictions.
