# Supplementary material for: Impact of sex and serum lipids interaction on working memory: A large‐scale brain networks study
Source: Brain Behav. 2023 May 10;13(7):e3054. doi: 10.1002/brb3.3054 (PMC10338845; doi:10.1002/brb3.3054)
Supplement: Supplementary file 1 — Figure S1. Spatial maps of 14 selected functional networks. Figure S2. Mediation analysis model. Figure S3. Using G*Power software, a post hoc analysis was performed to determine the relationship between statistical power and sample size, our samples could detect with adequate power (>95%) based on an alpha=0.05, indicating that our sample size was adequate. Table S1. Sex differences in the associations between triglyceride and inter‐network functional connectivity. Table S2. Sex differences in the associations between serum lipid and intra‐network functional connectivity. Table S3. Sex differences in the associations between triglyceride and inter‐network functional connectivity after additional adjustment for education level. Table S4. Sex differences in the associations between serum lipid and intra‐network functional connectivity after additional adjustment for education level. Table S5. Sex differences in the associations between triglyceride (TG) and inter‐network functional connectivity after matching the serum TG levels between male and female subjects. [file BRB3-13-e3054-s001.docx]

**Supplementary Materials**

**Supplementary Methods**

**Mediation analysis models**

Mediation analysis was performed using the PROCESS macro (Honey et al., 2009). In the mediation models (Figure S2), all paths were reported as unstandardized ordinary least squares regression coefficients, namely, total effect of X on Y (c) = indirect effect of X on Y through M (a × b) + direct effect of X on Y (c’). The significance analysis was based on 10,000 bootstrap realizations and a significant indirect effect is indicated when the bootstrap 95% confidence interval (CI) does not include zero. In the mediation analysis, only variables that showed a significant correlation with others were considered independent (serum lipids), dependent (cognitive functions), or mediating (intra- and internetwork functional connectivity) variables. Age, FD, and educational level were considered nuisance variables.

**Supplementary figures**


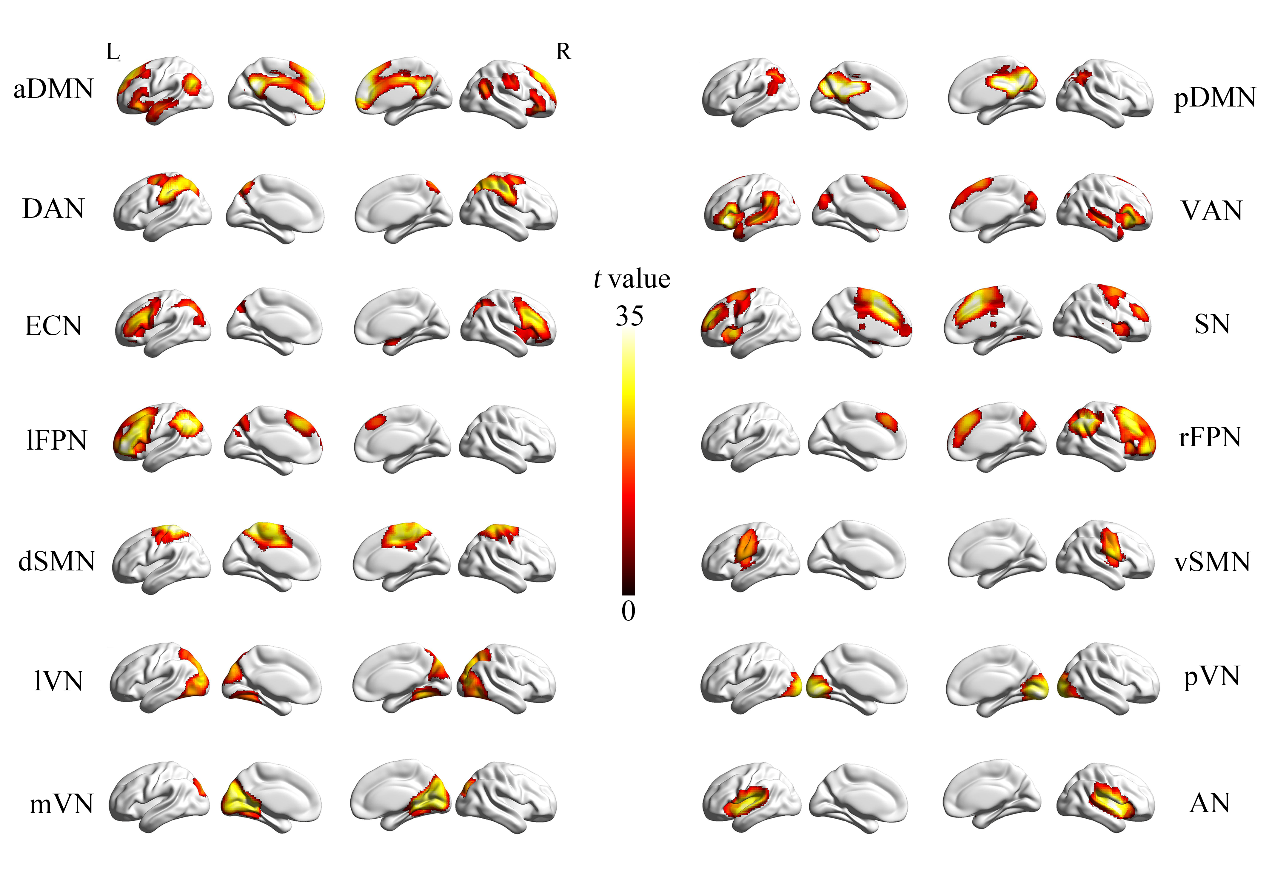


**Figure S1.** Spatial maps of 14 selected functional networks. The color scale represents *t* value. Abbreviations: aDMN, anterior default mode network; AN, auditory network; DAN, dorsal attention network; dSMN, dorsal sensorimotor network; ECN, executive control network; L, left; lFPN, left frontoparietal network; lVN, lateral visual network; mVN, medial visual network; pDMN, posterior default mode network; pVN, posterior visual network; R, right; rFPN, right frontoparietal network; SN, salience network; VAN, ventral attention network; vSMN, ventral sensorimotor network.


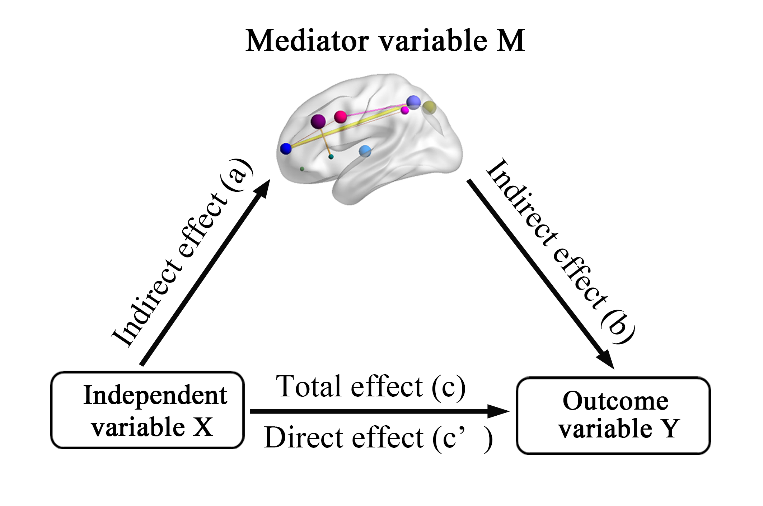


**Figure S2**. Mediation analysis model. All paths were reported as unstandardized ordinary least squares regression coefficients, namely, total effect of X on Y (c) = indirect effect of X on Y through M (a × b) + direct effect of X on Y (c’).


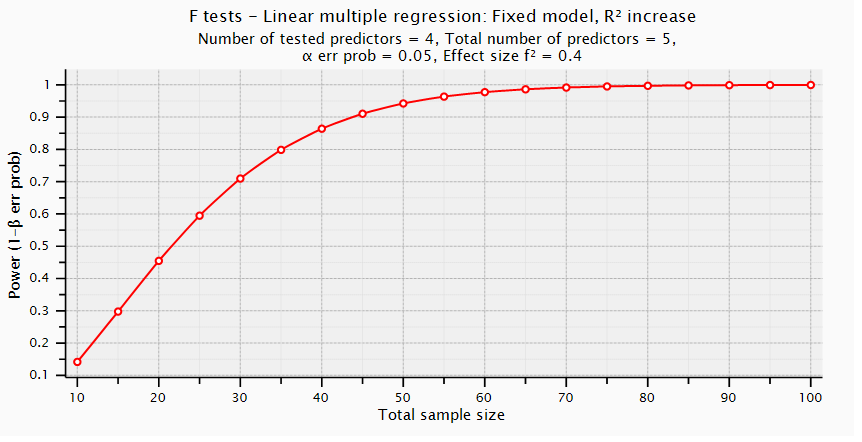


**Figure S3.** Using G*Power software, a post hoc analysis was performed to determine the relationship between statistical power and sample size, our samples could detect with adequate power (>95%) based on an alpha=0.05, indicating that our sample size was adequate.

**Supplementary tables**

**Table S1.** Sex differences in the associations between triglyceride and inter-network functional connectivity.

| Inter-network functional connectivity | *r*_males_ (*p*) | *r*_females_ (*p*) | *Z* value of sex comparison in *r* (*p*) | Cohen’s *q* |
| --- | --- | --- | --- | --- |
| ECN-dSMN | 0.011 (0.924) | -0.385 (< 0.001) | 2.56 (0.011) | 0.417 |
| ECN-mVN | -0.028 (0.808) | -0.452 (< 0.001) | 2.82 (0.005) | 0.459 |
| ECN-AN | 0.149 (0.193) | -0.299 (0.009) | 2.82 (0.005) | 0.459 |
| ECN-DAN | -0.053 (0.643) | -0.382 (< 0.001) | 2.15 (0.032) | 0.349 |

Abbreviations: AN, auditory network; dSMN, dorsal sensorimotor networks; DAN, dorsal attention networks; ECN, executive control network; mVN, medial visual networks. Cohen’s *q* (small effect: 0.1 < *q* < 0.3, intermediate effect: 0.3 < *q* < 0.5, large effect: *q* > 0.5) (Cohen, 1988).

**Table S2.** Sex differences in the associations between serum lipid and intra-network functional connectivity.

| Intra-network functional connectivity | *r*_males_ (*p*) | *r*_females_ (*p*) | *Z* value of sex comparison in *r* (*p*) | Cohen’s *q* |
| --- | --- | --- | --- | --- |
| **TC** |  |  |  |  |
| R-IPL in DAN | -0.326 (0.004) | 0.393 (< 0.001) | -4.63 (< 0.001) | 0.754 |
| R-MTG in lVN | 0.469 (< 0.001) | -0.214 (0.066) | 4.46 (< 0.001) | 0.726 |
| **LDL-C** |  |  |  |  |
| B-SFGmed in aDMN | 0.469 (< 0.001) | -0.381 (0.001) | 5.59 (< 0.001) | 0.910 |

Abbreviations: aDMN, anterior default mode networks; DAN, dorsal attention networks; TC, total cholesterol; R, right; IPL, inferior parietal; MTG, Middle temporal gyrus; lVN, lateral visual networks; LDL-C, low density lipoprotein cholesterol; B, bilateral; SFGmed, medial superior frontal gyrus. Cohen’s *q* (small effect: 0.1 < *q* < 0.3, intermediate effect: 0.3 < *q* < 0.5, large effect: *q* > 0.5) (Cohen, 1988).

**Table S3.** Sex differences in the associations between triglyceride and inter-network functional connectivity after additional adjustment for education level.

| Inter-network functional connectivity | *r*_males_ (*p*) | *r*_females_ (*p*) | *Z* value of sex comparison in *r* (*p*) | Cohen’s *q* |
| --- | --- | --- | --- | --- |
| ECN-dSMN | 0.035 (0.762) | -0.379 (0.001) | 2.67 (0.008) | 0.434 |
| ECN-mVN | -0.007 (0.955) | -0.448 (< 0.001) | 2.93 (0.003) | 0.475 |
| ECN-AN | 0.169 (0.142) | -0.307 (0.008) | 3.01 (0.003) | 0.488 |
| ECN-DAN | -0.041 (0.724) | -0.376 (0.001) | 2.18 (0.029) | 0.354 |

Abbreviations: AN, auditory network; dSMN, dorsal sensorimotor networks; DAN, dorsal attention networks; ECN, executive control network; mVN, medial visual networks. Cohen’s *q* (small effect: 0.1 < *q* < 0.3, intermediate effect: 0.3 < *q* < 0.5, large effect: *q* > 0.5) (Cohen, 1988).

**Table S4.** Sex differences in the associations between serum lipid and intra-network functional connectivity after additional adjustment for education level.

| Intra-network functional connectivity | *r*_males_ (*p*) | *r*_females_ (*p*) | *Z* value of sex comparison in *r* (*p*) | Cohen’s *q* |
| --- | --- | --- | --- | --- |
| **TC** |  |  |  |  |
| R-IPL in DAN | -0.331 (0.003) | 0.407 (< 0.001) | -4.78 (< 0.001) | 0.776 |
| R-MTG in lVN | 0.470 (< 0.001) | -0.230 (0.048) | 4.59 (< 0.001) | 0.744 |
| **LDL-C** |  |  |  |  |
| B-SFGmed in aDMN | 0.469 (< 0.001) | -0.442 (< 0.001) | 6.06 (< 0.001) | 0.984 |

Abbreviations: aDMN, anterior default mode networks; DAN, dorsal attention networks; TC, total cholesterol; R, right; IPL, inferior parietal; MTG, Middle temporal gyrus; lVN, lateral visual networks; LDL-C, low density lipoprotein cholesterol; B, bilateral; SFGmed, medial superior frontal gyrus. Cohen’s *q* (small effect: 0.1 < *q* < 0.3, intermediate effect: 0.3 < *q* < 0.5, large effect: *q* > 0.5) (Cohen, 1988).

**Table S5.** Sex differences in the associations between triglyceride (TG) and inter-network functional connectivity after matching the serum TG levels between male and female subjects.

| Inter-network functional connectivity | *r*_males_ (*p*) | *r*_females_ (*p*) | *Z* value of sex comparison in *r* (*p*) | Cohen’s *q* |
| --- | --- | --- | --- | --- |
| ECN-dSMN | 0.195 (0.114) | -0.391 ( 0.001) | 3.53 (< 0.001) | 0.611 |
| ECN-mVN | -0.007 (0.954) | -0.434 (< 0.001) | 2.65 (0.008) | 0.458 |
| ECN-AN | 0.156 (0.207) | -0.299 (0.014) | 2.70 (0.007) | 0.466 |
| ECN-DAN | 0.188 (0.127) | -0.371 (0.002) | 3.36 (< 0.001) | 0.580 |

Abbreviations: AN, auditory network; dSMN, dorsal sensorimotor networks; DAN, dorsal attention networks; ECN, executive control network; mVN, medial visual networks. Cohen’s *q* (small effect: 0.1 < *q* < 0.3, intermediate effect: 0.3 < *q* < 0.5, large effect: *q* > 0.5) (Cohen, 1988).

**References**

Cohen, J. (1988). The Statistical Power Analysis for the Behavioral Sciences. *Journal of the American Statistical Association, 2nd*(334).

Honey, C. J., Sporns, O., Cammoun, L., Gigandet, X., Thiran, J. P., Meuli, R., & Hagmann, P. (2009). Predicting human resting-state functional connectivity from structural connectivity. *Proceedings of the National Academy of Sciences of the United States of America, 106*(6), 2035-2040.
